# [Retracted] Altered expression of miR-92a correlates with Th17 cell frequency in patients with primary biliary cirrhosis

**DOI:** 10.3892/ijmm.2024.5476

**Published:** 2024-12-23

**Authors:** Dong-Yu Liang, Yan-Qiang Hou, Li-Jun Luo, Li Ao

Int J Mol Med 38: 131-138, 2016; DOI: 10.3892/ijmm.2016.2610

Following the publication of this paper, it was drawn to the Editor's attention by a concerned reader that several of the flow cytometric plots shown in Fig. 4 on p. 135 contained strikingly similar regions of data, although located in relatively different positions in various of the plots, to such an extent that it would have been difficult to have attributed these similarities to coincidence. After having performed an independent review of the data in the Editorial Office, the Editor of *International Journal of Molecular Medicine* has decided that this paper should be retracted from the Journal on account of a lack of confidence in the presented data. The authors were asked for an explanation to account for these concerns, but the Editorial Office did not receive a reply. The Editor apologizes to the readership for any inconvenience caused.

